# Cerebrospinal fluid opening pressure in relation to symptomatology and craniocervical anatomy in patients with myalgic encephalomyelitis/chronic fatigue syndrome

**DOI:** 10.3389/fmed.2026.1869714

**Published:** 2026-08-04

**Authors:** C. Jolley, B. Bragée, L. Soinne, H. Huhmar, H. Billing, B. Bertilson, P. Sjogren

**Affiliations:** 1 Kirk Kerkorian School of Medicine at the University of Nevada, Las Vegas (UNLV), Las Vegas, NV, United States; 2 Bragée Clinics, Stockholm, Sweden; 3 Department of Neurobiology, Care Sciences and Society, Karolinska Institutet, Huddinge, Sweden; 4 Karolinska University Hospital, Stockholm, Sweden

**Keywords:** CSF pressure, ICP, ME/CFS, MRI, symptomathology

## Abstract

**Background:**

Myalgic encephalomyelitis/chronic fatigue syndrome (ME/CFS) is a debilitating disease that affects millions worldwide. Its cause remains unknown, although many physiological mechanisms are impaired. Structural abnormalities in the craniocervical region seem to be more prevalent in patients with ME/CFS, with a possible impact on intracranial pressure (ICP) and lumbar puncture (LP) measurements. This study aimed to quantify the occurrence of elevated cerebrospinal fluid (CSF) opening pressure and a possible association with clinical symptoms and radiological variables in craniocervical segments in a cohort of patients with ME/CFS.

**Methods:**

This study included 34 patients previously diagnosed with ME/CFS as part of a larger case–control study, for whom LP was performed only in the case group. Key measurements included age, sex, opening pressure from LP, symptomatic relief post-LP, and radiological variables with potential influence on CSF dynamics. Individuals were stratified into two groups according to opening pressure (OP) measurements using 20 cmH_2_O as the cutoff value, with subsequent statistical comparisons.

**Results:**

Overall, the majority of participants reported moderate disease severity (65%) and concomitant self-reported low quality of life. The median CSF opening pressure was 18 (IQR 8) cmH_2_O, ranging from 11 to 29.5. Opening pressure stratification showed that 21 of the 34 cases (62%) had an OP measurement of <20 cmH_2_O (low-OP group) and 13 cases (38%) had an OP measurement of ≥20 cmH_2_O (high-OP group). The two OP groups did not differ significantly in baseline characteristics, including disease severity and quality of life. However, for certain radiological measurements, the high-OP group presented with measures reflecting a wider spinal canal, including a significantly larger foramen magnum diameter (33.9 mm), which was higher than the measures in the low-OP group (31.7 mm). Estimates of symptom relief 1–4 days post-LP were significantly higher among patients in the high-OP group (80%) than among those in the low-OP group (19%).

**Conclusion:**

This study suggests that a proportion of patients with ME/CFS have elevated CSF opening pressures and may experience symptom relief following CSF subtraction. Our data indicate that some anatomical structures in the craniocervical area may be related to CSF pressure, although interpretation is challenging and merits further investigation.

## Introduction

Myalgic encephalomyelitis/chronic fatigue syndrome (ME/CFS) is a chronic, multisystem disease (1, 2). Its hallmark symptom is post-exertional fatigue that is unrelieved with rest following activities that require cognitive, emotional, and/or physical effort (2, 3). Patients with ME/CFS may also experience a mixture of other symptoms such as orthostatic intolerance, pain hypersensitivity, headaches, muscle pain, and neurocognitive impairments (e.g., brain fog) (2, 4, 5). ME/CFS affects millions of people worldwide and has an estimated prevalence of between 0.52 and 1.04% in the United States (3). ME/CFS is more common among women and middle-aged individuals (3, 6). Many patients with ME/CFS also suffer from comorbidities and report severe impairments that disrupt their education, family and social life, emotional wellbeing, and ability to perform daily living tasks (7).

The pathophysiology and etiology of ME/CFS remain relatively unknown (8, 9). Research aimed at determining impaired physiological mechanisms has revealed irregularities in the immune, endocrine, metabolic, and autonomic nervous systems (1, 10). The most widely accepted etiologic theory has existed for more than three decades and proposes that the disease develops following a viral infection (11). Another theory suggests that there is an underlying genetic component that makes individuals more susceptible to developing the disease (11). While there is an association between disease onset, previous viral infections, and genetic susceptibilities, these theories have discrepancies and lack full consensus (12). Recognizing the central nervous system (CNS)-related symptomatology of ME/CFS, several research groups have proposed that its development is facilitated by structural abnormalities in the craniocervical region. It is hypothesized that these abnormalities may impede the natural flow of blood and/or cerebrospinal fluid (CSF), thus causing alterations in CNS activity (13–15). In one specific study, researchers found a disproportionate number of obstructions in the craniocervical region in patients with ME/CFS (13). They also found that patients with ME/CFS were more likely to have signs of intracranial hypertension, which may be of particular relevance to the pathophysiology of ME/CFS. Interestingly, typical symptoms of ME/CFS overlap considerably with those of idiopathic intracranial hypertension (IIH), which is characterized by an intracranial pressure (ICP) of >20 cmH_2_O. Previous research has shown that patients with ME/CFS frequently exhibit signs of elevated CSF pressure and that typical symptoms of ME/CFS improve significantly following CSF subtraction (13, 16, 17).

**Figure 1:**
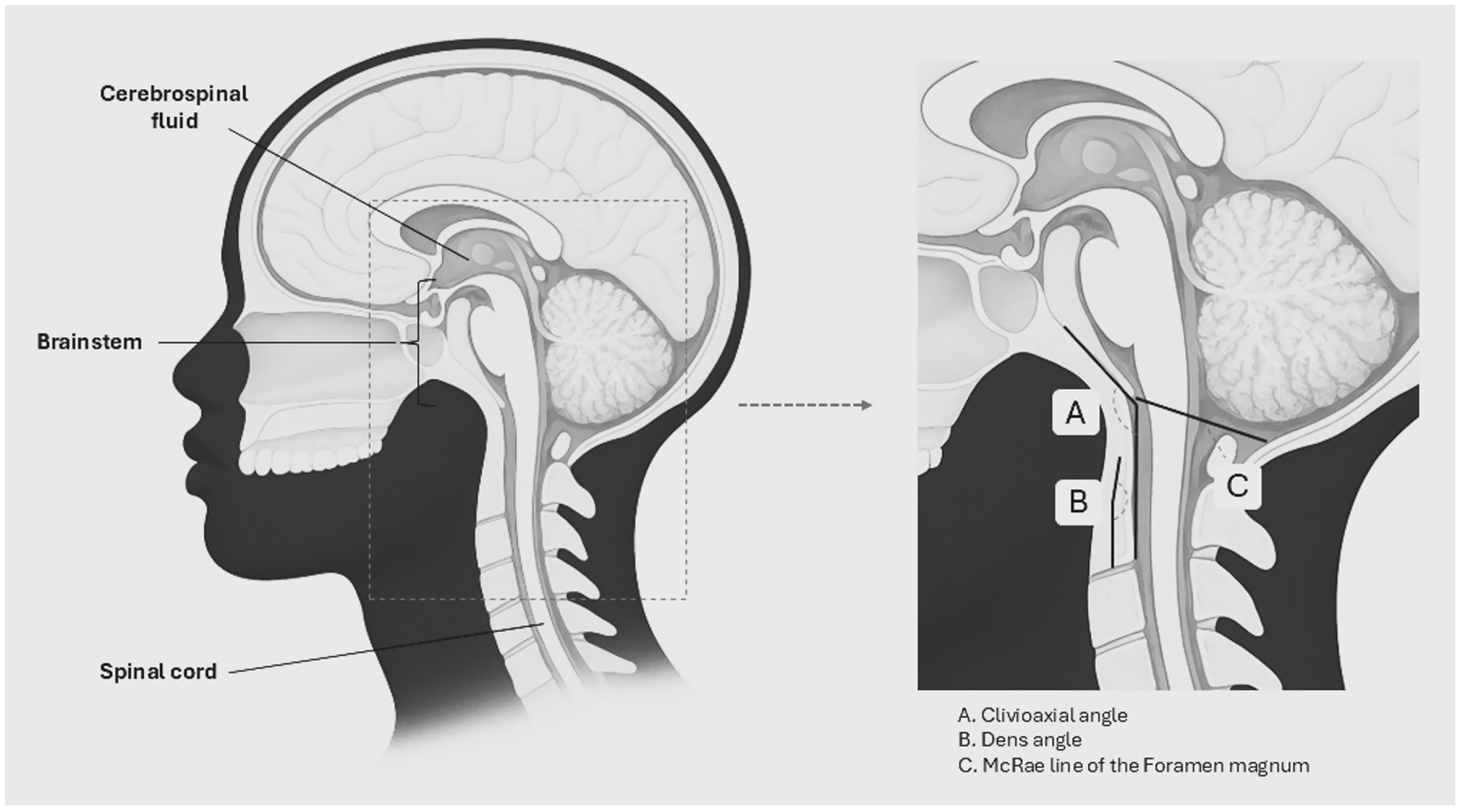
Midsagittal view of the skull, brain, and upper cervical spine with the brainstem area and the area surrounding the craniocervical junction augmented on the right panel. The clivo-axial angle **(A)**, the dens angle **(B)**, and the McRae line of the foramen magnum **(C)** are highlighted to visualize selected measures discussed in this study. The illustration should be considered schematic, as being constructed with AI assistance (Copilot and Microsoft) and not as anatomically accurate.

Craniocervical anatomy is complex and involves many structures that may influence CSF dynamics. One such structure is the transition area between the brain and the cervical spine, i.e., the foramen magnum, of which the McRae line represents a key measurement (Figure 1). Structures such as the cerebellar tonsils and the foramina of Luschka and Magendie are important for CSF flow (16, 17). Connective tissue disorders such as Ehler–Danlos syndrome (EDS) have been reported in patients with ME/CFS and may alter structures near the craniocervical junction, which may contribute to abnormal pressure dynamics (18–20). To estimate the presence of such abnormalities contributing to CSF flow irregularities, measurements such as the dens angle and clivo-axial angle (CXA) can be informative (Figure 1).

This study aimed to examine the occurrence of elevated cerebrospinal fluid opening pressure and its association with clinical symptoms and craniocervical radiological findings in a cohort of patients with ME/CFS.

## Methods

This retrospective, cross-sectional study used data collected from a ME/CFS specialty clinic in Stockholm, Sweden, from 2021 to 2022. Ethical approval for the study was granted by the Swedish Ethical Review Authority (#2019–03510 and #2022–06786-02), and all participants provided written informed consent.

A total of 34 patients were included in the present study, selected from a larger case–control study including 40 patients diagnosed with ME/CFS according to the Canadian Consensus Criteria (CCC), who were free from known spinal disease, and 41 healthy gender and age-matched controls. The ME/CFS diagnosis was verified at study entry by a specialist physician (BB). The current analyses included only the cases, as ethical constraints prohibited LP in healthy participants. Furthermore, LP could not be performed in 6 of the patients, resulting in 34 cases being included in the present analyses. The inability to perform LP in these six patients was due to unsuccessful penetration of the spinal canal following two to three attempts. The six patients who did not complete LP did not differ significantly from the 34 patients who completed the procedure in terms of disease severity, disease duration, or overall symptomatology (all *p* > 0.30).

LP was performed on patients fulfilling the following inclusion criteria: magnetic resonance imaging (MRI) assessed by a third-party radiologist showing cerebellar tonsil herniation through the foramen magnum of <5 mm; no bleeding disorder (International normalized ratio (INR) for blood clotting > 1.8; Trombocyte count < 50); and/or underwent no treatment with antithrombotic drugs (e.g., Coumadin), new direct-acting oral anticoagulants (NOACs), antiplatelet inhibitors, or high-dose heparin. The LP was performed by two specialist physicians (BB and LS) in a standardized manner, with the patient in the lateral recumbent position, legs relaxed, and the head in a neutral alignment. A 9-cm, 22-gauge needle was subsequently used to enter the subarachnoid space at the levels of L3–L4, L4–L5, or L5–S1. Upon accessing the subarachnoid space, a manometer was attached to the spinal needle via tubing to measure the CSF opening pressure. The CSF pressure was noted by the clinician after the apparatus reached equilibrium. After equilibrium was reached, 10 mL of CSF was collected for subsequent biobanking. Patients were asked to rate their symptoms (headache, brain fog, pain, and fatigue) on a 10-point Likert scale before LP and over a 1-h period post-LP. A delta change in symptoms was calculated by comparing the ratings collected at 60 min post-procedure with the ratings obtained prior to the procedure. Patients were contacted by the clinic’s nurse via telephone 1–4 days following LP for an unstructured interview regarding subjective post-visit changes in their overall health status and ME/CFS-specific symptoms, such as brain fog and pain/headache. In total, follow-up occurred on post-LP day 1 (*n* = 21), day 2 (*n* = 3), day 3 (*n* = 2), and day 4 (*n* = 3). Five participants were not reachable within 4 days post-visit. A self-reported significant improvement in overall health status at this follow-up was classified as post-LP symptom relief after CSF subtraction. Closing pressure was not determined. Adverse post-visit changes were also noted at follow-up, including aggravated headache and post-exertional malaise (PEM).

### General characteristic variables

Age, sex, BMI, height, systolic and diastolic blood pressure, C-reactive protein (CRP), serum cortisol, thyroid-stimulating hormone (TSH), general joint hypermobility, time since diagnosis, disease severity (e.g., mild, moderate, and severe), gait speed, quality of life, and physical function were assessed. General joint hypermobility was evaluated using the Beighton score, with a score of ≥5 on a 0–9 scale indicating hypermobility (21). Disease severity was classified as mild, moderate, or severe according to the patient’s responses to the clinic’s ME/CFS symptom questionnaire (22). Patients with very severe ME/CFS were excluded from the study. Gait speed was assessed by timing a 10-m walk (s), with participants instructed to walk at their usual high speed that would not normally elicit complications afterward. Physical function and quality of life were measured via the RAND 36-Item Health Survey and EQ-5D questionnaire, respectively.

### Magnetic resonance imaging (MRI) measurements

MRI scans of the craniocervical region were completed using 1.5 Tesla scanners at radiological facilities within the Stockholm region. MRI scans were obtained with patients in a supine position. Scans were evaluated independently by a radiologist to verify abnormalities affecting LP procedures, such as enlarged ventricles or obliterated basal cisterns. Images from MRI were further used in the current evaluation of craniocervical structural anatomy, as described below. The MRI scans were measured by a neuroradiologist and a medical student using built-in tools in Sectra® software.

To measure the foramen magnum diameter, cerebellar tonsil distance from the McRae line, the dens angle, and CXA, the midsagittal view from T1-weighted scans of the craniocervical region was used. Foramen magnum diameter was measured by annotating the scan from the basion to the opisthion. The annotation also served as a reference point to measure the cerebellar tonsil distance from the McRae line. Positive values indicate that the tonsils are positioned above the foramen magnum (rostrally) in the posterior fossa, while negative values signify that the tonsils are located below the foramen magnum (caudally). During this measurement, patients were evaluated for Chiari malformation again to verify measurements completed by the third-party radiologist. The dens angle was measured as the angle between the C2 vertebral body, the atlantoaxial joint midpoint, and the most superior aspect of the odontoid process’s (dens) midpoint. The CXA was measured as the angle between the long axis of the clivus and the posterior aspect of the C2 vertebral body (see Figure 1).

The midsagittal view from T2-weighted scans was used to assess the presence of olisthesis (anterolisthesis and retrolisthesis), abnormal cervical spine shape, disk height deviation, spinal canal and cord diameter at C2–C3, and the narrowest point of the spinal canal. Abnormal cervical spine shape designation was defined as the deviation of the spine from the normal lordotic curvature, such as being flat or kyphotic. Deviation in disk height was assessed by determining whether the intervertebral disks followed the non-pathological pattern of increasing thickness in the cranial-to-caudal direction (23). The intervertebral level (e.g., C2–C3 and C3–C4) that corresponded to the spinal canal’s narrowest point was first estimated visually on the T2-weighted midsagittal view and then confirmed using the software’s measurement tools. After this point was identified, the midsagittal view was positionally synchronized with T2-weighted transverse (axial) scans. The transverse spinal canal and cord areas at the narrowest point were then measured.

The anteroposterior (AP) and lateral diameters, as well as the spinal canal and cord areas, were assessed at the C3–C4 level using the transverse (axial) view on T2-weighted MRI for each respective patient. Canal-to-cord area ratios were calculated at the C3–C4 intervertebral level and at the narrowest point in the spinal canal. These calculations were based on a ratio of <1.25, which is a sensitive marker of canal stenosis that may increase the risk of degenerative cervical myelopathy (24, 25). Intervertebral disk protrusion (i.e., mild, moderate, and severe) was assessed using T2-weighted images. Mild disk protrusion was assessed as an intervertebral disk protrusion into the spinal canal space without contacting the cord. Moderate disk protrusion was assessed as a protrusion completely obstructing the spinal canal without affecting the cord. Severe disk protrusion was assessed as a disk protrusion into the spinal cord or a nerve root.

The optic nerve sheath diameter (ONSD) was measured 3 mm from the midline of the optic nerve’s inner limit along the axis of the posterior chamber. Eyeball transverse diameter (ETD) was measured from the left to the right inner edges of the retina in the posterior chamber where the diameter was greatest. Both ONSD and ETD were measured using T2-weighted MRI axial scans. The ONSD/ETD ratio was subsequently calculated for each patient following measurement. A normal ONSD/ETD ratio was considered to be 0.19 ± 0.02. An abnormal ratio was considered to be >0.25, which may be related to abnormal intracranial pressure and intracranial hypertension (IH) (26).

### Statistical analysis

Participants were stratified according to opening pressure measurements using 20 cmH_2_O as the cutoff, resulting in a low-pressure group (low-OP of <20 cmH_2_O) and a high-pressure group (high-OP of ≥ 20 cmH_2_O). A spreadsheet with all patient data was used to organize data by group. Shapiro–Wilk tests were run for each continuous variable to assess for normal distribution. If values were normally distributed, the mean and standard deviation (SD) were calculated; otherwise, the median and interquartile ranges were reported. Categorical values (e.g., female sex and general joint hypermobility) were reported as n (%). *p*-values were then calculated to assess statistical differences between the two stratified groups (i.e., <20 cmH_2_O or ≥20 cmH_2_O). T-tests or Mann–Whitney U-tests were used based on the distribution of continuous variables, and two-sample proportion or independent chi-squared tests were used for categorical variables. Due to the exploratory nature of the study, the results were not corrected for multiple comparisons.

## Results

### Whole study group

Characteristics of the 34 patients with ME/CFS included in our analysis are depicted in Table 1, with the majority of participants having moderate disease severity (65%) and a concomitant self-reported low quality of life (median 30 on a 0–100 scale). Participants were, on average, 46 years old, female (77%), and slightly overweight (mean BMI 25.2), with a high occurrence of general joint hypermobility (41%). Levels of serum cortisol, TSH, and CRP were all within the normal range. The median CSF opening pressure was 18 (IQR 8) cmH_2_O, ranging from the lowest detected value of 11 cmH_2_O to the highest detected value of 29.5 cmH_2_O (Table 2). Classical symptoms of ME/CFS (headache, brain fog, pain, and fatigue) were essentially unchanged at the group level within 60 min post-LP. However, 11 (32%) of the patients reported subjective symptom relief 1–4 days post-LP. Among the 11 patients who experienced symptom relief, 7 reported significant improvements in brain fog and 5 reported significant improvements in pain/headache. In fact, the health status of 3 of the 11 patients improved to a degree such that they felt almost completely recovered and could participate in various ordinary day-to-day activities (such as shopping or spending time with their kids outside). The improvements remained for 4–6 days post-LP and then gradually vanished. Given the effort required by patients throughout the visit, which included multiple tests and procedures (e.g., LP), a limited fraction of patients experienced adverse effects post-visit. Follow-up data were available in 26 individuals, of whom 5 reported aggravated headache and 12 reported moderate or severe PEM 1–4 days post-visit. Of note, the re-evaluation of the radiological images revealed three patients with cerebellar tonsils lying 5 mm below the McRae line.

**Table 1:** Characteristics of the included patients with ME/CFS who underwent lumbar puncture.

Characteristics	All		Stratified for opening pressure			
	*n*	Values	*n* per group	<20 cmH_2_O	≥20 cmH_2_O	*p*-value
Age (years), mean (SD)	34	46 (11)	21/13	47 (11)	43 (12)	0.30
Female, *n* (%)	34	26 (77)	21/13	17 (81)	9 (69)	0.43
BMI (kg/m^2^), median (IQR)	34	25.2 (7.0)	21/13	23.7 (4.6)	28.4 (10.1)	0.40
Height (cm), mean (SD)	34	170 (10)	21/13	169 (9)	172 (12)	0.44
Systolic BP (mmHg), mean (SD)	31	121 (16)	20/11	120 (19)	122 (11)	0.48
Diastolic BP (mmHg), mean (SD)	30	77 (10)	19/11	77 (10)	78 (10)	0.75
CRP (mg/L), median (IQR)	27	1 (2)	17/10	1 (1)	1.5 (2.8)	0.61
Serum cortisol (nmol/L), mean (SD)	28	407 (123)	17/11	371 (101)	462 (139)	0.08
Serum TSH (mIU/L), mean (SD)	28	2.2 (1.1)	17/11	1.9 (1.0)	2.6 (1.3)	0.17
General joint hypermobility *n* (%)	34	14 (41)	21/13	8 (38)	6 (46)	0.64
Time since diagnosis in months, median (IQR)	34	28 (20)	21/13	29 (23)	28 (14)	0.89
Disease severity	34		21/13			
Mild *n* (%)		3 (9)		2 (10)	1 (8)	0.86
Moderate *n* (%)		22 (65)		13 (62)	9 (69)	0.66
Severe *n* (%)		9 (27)		6 (29)	3 (23)	0.72
Gait speed in m/s, mean (SD)	32	1.2 (0.3)	20/12	1.3 (0.3)	1.2 (0.3)	0.58
Quality of life 0–100, median (IQR)	33	30 (15)	21/12	31 (12)	28 (7)	0.16
Physical function 0–100, median (IQR)	33	35 (35)	21/11	35 (20)	22 (48)	0.33

Values are presented as mean (SD) if normally distributed, otherwise median (IQR). Categorical variables are given in *n* (%). *P*-values represent statistical differences between the groups stratified according to opening pressure measurements, i.e., above/below 20 cmH2O, and are calculated using a t-test or Mann–Whitney *U*-test for continuous variables and a two-sample proportion test or independent chi-squared test for categorical variables. BMI, body mass index; BP, blood pressure; CRP, C-reactive protein, TSH, thyroid-stimulating hormone.

**Table 2:** Measures of cerebrospinal fluid pressure and symptoms in relation to the lumbar puncture procedure in the included patients with ME/CFS.

Variables	All		Stratified for opening pressure			
	*n*		*n* per group	<20 cmH_2_O	≥20 cmH_2_O	*P*-value
Symptom severity in relation to lumbar puncture procedure, Likert scale 0–10^a^						
Headache before LP, median (IQR)	29	2 (5)	16/13	2 (3.5)	3 (5)	0.75
∆ Headache, mean (SD)	29	−0.9 (1.9)	16/13	−0.8 (2.1)	−1.1 (1.6)	0.59
Brain fog before LP, mean (SD)	29	4.7 (2)	16/13	4.4 (1.9)	5 (2.1)	0.42
∆ Brain fog, median (IQR	29	−1 (3)	16/13	−1 (2.3)	−1 (2)	0.66
Pain before LP, mean (SD)	29	4.3 (2.3)	16/13	4.3 (2.0)	4.4 (2.8)	0.89
∆ Pain, median (IQR)	28	−1 (2)	16/12	−1 (2)	−1 (5)	0.21
Fatigue before LP, median (IQR)	29	7 (3)	16/13	7 (2.3)	7 (3)	0.69
∆ Fatigue, median (IQR)	29	−1 (3)	16/13	−0.5 (2.3)	−1 (2)	0.37
Symptom relief post visit, *n* (%) ^b^	26	11 (42)	16/10	3 (19)	8 (80)	0.004

LP, lumbar puncture. Values are presented as mean (SD) if normally distributed, otherwise median (IQR). Categorical variables are given in *n* (%). *P*-values represent statistical differences between the groups stratified according to opening pressure measurements, i.e., above/below 20 cmH2O, and are calculated using a *t*-test or a Mann–Whitney *U*-test for continuous variables and a two-sample proportion test or an independent chi-squared test for categorical variables.

^a^ Symptom severity was reported prior to the initiation of LP (before LP), and changes in symptoms during the following hour (∆-values) were reported on a Likert scale of 0–10.

^b^ Self-reported improvements in overall health status at follow-up 1–4 days following LP-visit.

### Group analyses

Patients were stratified according to CSF opening pressure and, among the 34 patients included, 21 (62%) had CSF opening pressure of <20 cmH_2_O (low-OP), while 13 (38%) had CSF opening pressure of ≥20 cmH_2_O (high-OP). The two groups (low-OP vs. high-OP) did not differ significantly in baseline characteristics, as presented in Table 1. As shown in Table 2, no group differences were detected in symptom changes within 60 min post-LP, but a significantly higher proportion of patients in the high-OP group (80%) experienced subjective symptom relief 1–4 days post-LP compared with 19% in patients in the low-OP group (*p* = 0.004). Values for post-visit change were missing for 8 individuals. Sensitivity analysis—assuming the eight missing individuals were non-responders (i.e., no symptom change)—did not affect the outcome (*p* < 0.01). Next, we examined whether OP groups (i.e., low vs. high-OP) differed in terms of selected measures of craniocervical anatomy derived from MRI scans. The radiological variables were selected for their potential influence on CSF dynamics (Table 3). An ONSD/ETD ratio of >0.25, which is frequently used as a proxy measure of elevated intracranial pressure, did not differ significantly between the two groups. Furthermore, measures of spinal canal width tended to be higher in the high-OP group, as indicated by a wider foramen magnum (mean 33.9 vs. 31.7 mm, *p* = 0.029) and a wider transverse spinal canal area at the narrowest point (mean 210 vs. 185 mm, *p* = 0.06). No differences between the groups were observed for spinal cord measurements, cervical spine shape (e.g., flat or kyphotic shape), or cerebellar tonsil position from the McRae line. The occurrence of olisthesis was significantly higher in the high-OP group (31%) than in the low-OP group (5%), *p* = 0.037. Finally, compared to low-OP individuals, patients in the high-OP group had a smaller dens angle (median 172 vs. 176 degrees, *p* = 0.019) and a tendency toward a larger CXA (median 154 vs. 149 degrees, *p* = 0.052). Importantly, the detected differences between the low-OP and high-OP groups for certain radiological measures, such as a wider foramen magnum, were not explained by differences in body composition. In fact, the foramen magnum diameter and measures of spinal canal width were unrelated to body composition measurements, such as BMI and length (all *p* > 0.20, data not shown).

**Table 3:** Selected radiological measurements derived from magnetic resonance imaging of patients with ME/CFS who underwent lumbar puncture.

Measurements	All	Stratified for opening pressure		
		<20 cmH_2_O	≥20 cmH_2_O	*P*-value
	*n* = 34	*n* = 21	*n* = 13	
Unilateral ONSD/ETD > 0.25, *n* (%) ^a^	21 (62)	12 (57)	9 (69)	0.48
Cerebellar tonsil distance above/at/below the McRae line (mm), mean (SD)	0.8 (2.7)	1.2 (2.5)	0.1 (3)	0.30
Foramen magnum diameter (mm), mean (SD)	32.5 (3.4)	31.7 (3.8)	33.9 (2.0)	0.029
Dens angle (degrees), median (IQR)	174 (6)	176 (4)	172 (4)	0.019
Clivo-axial angle (degrees), median (IQR)	151 (8)	149 (11)	154 (8)	0.052
Olisthesis present, *n* (%)	5 (15)	1 (5)	4 (31)	0.037
Abnormal shape of cervical spine, *n* (%)	12 (35)	7 (33)	5 (38)	0.76
Disk height deviation present, *n* (%)	10 (29)	8 (38)	2 (15)	0.16
Severe disk protrusion present, *n* (%)	12 (35)	8 (38)	4 (31)	0.66
Sagittal spinal canal diameter at C2–C3 (mm), mean (SD)	13.3 (1.3)	13.2 (1.3)	13.3 (1.4)	0.81
Sagittal spinal cord diameter at C2–C3 (mm), median (IQR)	7 (1.3)	7 (1.6)	7 (0.3)	0.83
AP spinal cord diameter at C3–C4 (mm), median (IQR)	7 (0.5)	7 (0.9)	7 (0.2)	0.48
Lateral spinal cord diameter at C3–C4 (mm), median (IQR)	13 (2)	13 (2)	13 (2)	0.97
Transverse spinal cord area at C3–C4 (mm^2^), median (IQR)	74.0 (13.4)	72.7 (13.0)	76.9 (12.9)	0.94
Transverse spinal canal area at C3–C4 (mm^2^), mean (SD)	222.1 (36.8)	220.6 (35.6)	224.8 (40.1)	0.76
Canal-to-cord-area ratio at C3–C4, median (IQR)	2.8 (0.4)	2.9 (0.4)	2.7 (0.5)	0.78
Transverse spinal cord area at the most narrow point (mm^2^), mean (SD)	70.0 (14.1)	67.9 (16.3)	73.5 (8.9)	0.21
Transverse spinal canal area at the most narrow point (mm^2^), mean (SD)	194.5 (45.4)	184.9 (48.3)	209.8 (37.0)	0.060
Canal-to-cord-area ratio at the most narrow point, median (IQR)	2.7 (0.9)	2.5 (1.2)	2.7 (0.3)	0.43
Chiari malformation type 1, *n* (%)	3 (8.8)	1 (4.8)	2 (15.4)	0.289

Values are presented as mean (SD) if normally distributed, otherwise median (IQR). Categorical variables are given in *n* (%). *P*-values represent statistical differences between the groups stratified according to opening pressure measurements, i.e., above/below 20 cmH2O, and are calculated using a *t*-test or Mann–Whitney *U*-test for continuous variables and a two-sample proportion test or independent chi-squared test for categorical variables.

^a^ Presence of a ratio of >0.25 for optic nerve sheath diameter (ONSD) in relation to eyeball transverse diameter (ETD) for at least one eye, commonly used as a proxy measurement for elevated intracranial pressure.

## Discussion

In this cross-sectional study of 34 clinically verified cases of ME/CFS, we highlight the existence of elevated CSF pressure among patients and present the relationship between CSF pressure and MRI-derived craniocervical anatomy in this patient group. Our data reveal that a large portion of the patients (38%) had elevated CSF pressure measurements, as measured by lumbar puncture, and that a majority of those patients experienced symptom relief following CSF subtraction. In some cases, the reported symptom relief was associated with a remarkable but transient change in health status. The radiological assessment was, however, unable to identify any clear structural abnormalities that could explain the elevated CSF pressure. In fact, patients with high CSF pressure had a wider foramen magnum and a tendency toward a wider spinal canal than patients with low CSF pressure, and the potential implication of these findings warrant investigation in forthcoming studies.

Elevated ICP may contribute to symptoms or disease phenotype in a subgroup of patients diagnosed with ME/CFS (13, 27, 28). Our results are consistent with previous studies showing that elevated ICP is common in patients with ME/CFS (13, 28, 29). Several theories exist to explain the prevalence of elevated ICP in this patient group. One proposed model suggests that cranial venous obstructions induce alterations in intracranial pressure, leading to conditions like ME/CFS or IIH (30). However, elevated ICP may also be a consequence of impaired lymphatic and glymphatic clearance, leading to toxic buildup, neuroinflammation, and impaired functionality (15). Furthermore, structural abnormalities in the craniocervical region seem to be common in patients with ME/CFS, indicating that inherited and/or acquired structural abnormalities may influence CSF flow and ICP (13). It is also possible that diseases such as ME/CFS and IIH may occur simultaneously, with distinct pathophysiologic processes that cause symptoms non-specific to ME/CFS, such as fatigue, brain fog, and pain, to develop. ME/CFS may also be one of the manifestations of IIH. In a 2017 article, Higgins et al. focused on this idea and questioned whether they were different manifestations of an intracranial pressure disorder as evidenced by similar core symptoms such as fatigue and headache (31). Whether ME/CFS is one of the manifestations of a pressure/volume-related disorder, such as IIH, or whether they occur concurrently, these possible associations may explain the responsiveness to CSF subtraction among the patients with elevated ICP in the current study. Future studies are needed to investigate each of these possible hypotheses and why a possible subgroup sensitive to CSF removal exists in patients with ME/CFS.

The subtraction of CSF that results in symptomatic relief in a subgroup of our participants is of clinical interest. Approximately one-third of our patients experienced symptomatic relief following CSF subtraction. Patients with high OP reported symptom relief more frequently than those with low OP. Symptom relief following LP has been reported in this patient group by others. In their 2013 study, Higgins et al. found significant symptom improvement in response to spinal fluid subtraction via LP in all 5 patients with pressures greater than 20 cmH_2_O (28). Interestingly, 80% of their patients with normal CSF pressures (i.e., ≤20 cmH_2_O) also experienced symptom relief (28). Among our participants, only 14% of the patients in the low-OP group experienced symptomatic relief post-LP. Still, the occurrence of symptom relief following CSF subtraction in a portion of patients with no apparent pressure abnormalities is of interest and may imply that a subgroup of ME/CFS patients, independent of CSF opening pressure, is particularly sensitive to alterations in CSF dynamics. Whether this reflects compartmentalization of CSF remains elusive, although it is a possibility. How CSF subtraction influences CSF flow with subsequent improvements in ME/CFS symptoms remains open for speculation. The CSF volume extracted in our study was limited (10 mL), and whether this subtraction influenced CSF pressure is undetermined (as closing pressure was not quantified). Furthermore, the limited quantity of CSF extracted should have been replenished within the following 24 h, given normal CSF production. Still, symptom relief was evident in a subgroup of patients several days after CSF subtraction, which may indicate some intermediary mechanisms responsible for mediating symptom relief in relation to changes in CSF volume. However, we cannot rule out that other factors besides CSF subtraction influenced the self-reported improvement post-visit, although we lack indications of other such contributing factors. In fact, our participants were generally surprised by their health status at follow-up, considering the effort associated with the visit. Among responders, i.e., those with symptomatic improvements post-visit, relief of brain fog, and improved headache were frequently reported, and one patient referred to the CSF subtraction as “nose spray for her brain.”

To obtain further insights into possible structural abnormalities that may coincide with our findings, we evaluated a selection of MRI-derived datapoints in our patients. These exploratory analyses detected three radiological variables that differed significantly between the two OP groups, while two other variables showed a trend toward significance. For organizational purposes, these five variables can be separated into three categories: *i) structural* (i.e., foramen magnum diameter and transverse spinal canal area at the narrowest point in the cervical spine), *ii) spinal alignment* (i.e., dens angle and CXA), and *iii) spinal alignment with structural properties* (i.e., olisthesis). Regarding the structural variables, the foramen magnum diameter and the spinal canal area measurements were greater in the high-OP group, although neither group had average diameters considered pathological. This observation was unexpected, considering that a wider spinal canal would, intuitively, be related to improved CSF flow and lower OP. The foramen magnum represents a critical anatomical landmark because it marks the transition from the intracranial compartment to the spinal canal and contains structures such as the lower end of the medulla oblongata, spinal accessory nerve, and vertebral arteries (32). To the best of our knowledge, no previous studies have described associations between foramen magnum diameter, measures of spinal canal width, and CSF pressure. A mechanism for the observed relation between a wider spinal canal and higher opening pressure remains unclear, although it may reflect changes in compliance influencing the canal’s buffering ability in response to raised ICP (33). The radiological variables reflecting spinal alignment (i.e., dens angle and CXA) also yielded interesting findings, with a lower dens angle and a higher CXA in the high-OP group. The CXA is a measure of craniocervical instability and has a normal range of 145 to 160 degrees (20). The dens angle reflects retroflexion (posterior inclination) of the odontoid process (dens), and a previous study has suggested disrupted CSF flow as a consequence of dens retroflexion (i.e., lower dens angle) (34). Finally, olisthesis (with both alignment and structural elements) is believed to occur due to a variety of mechanisms, including facet joint and intervertebral disk degeneration, leading to symptoms such as neck pain and myelopathy (35). In our study, olisthesis was more prevalent among patients in the high-OP group. Previous research has shown that cervical olisthesis is associated with reduced volume within the spinal compartment, with a potential impact on OP measurements (36).

Overall, and based on our radiological examinations, we could not identify any specific deviation in craniocervical anatomy that explained alterations in OP among our individuals. We cannot exclude a possible distortion in spinal orientation caused by the supine positioning during MRI scanning, which may have hampered the interpretation of our findings. Furthermore, determinants of CSF dynamics involve complex mechanisms and factors not captured in the present study. One such factor may be measures of fluid homeostasis, including hormones such as antidiuretic hormone (ADH), and its subsequent effect on fluid osmolality. Disrupted fluid homeostasis with impaired ADH secretion was recently described in this patient group, which may influence CSF dynamics (37).

The limitations of our study include the sample size and generalization, the patient follow-up method post-LP, the lack of a control sample of individuals without ME/CFS, the cross-sectional design of MRI assessments, and the representativeness of ICP based on LP and MRI findings in the supine position. Our participants were representative of the patient population in terms of gender and age and were not recruited based on certain phenotypic features, but a larger sample size would have ensured greater generalizability of our findings. The LP approach is valuable due to its accessibility, although it may not reflect dynamic changes in ICP, such as those influenced by the time of day, patient positioning, and history of headache (38). A standardized amount of CSF was subtracted from each patient, but the closing pressure was not determined. Thus, we were unable to estimate the effect that CSF subtraction had on CSF pressure and whether any differences existed between the two groups. Furthermore, anatomical features that create obstructions in the craniocervical region may cause discrepancies between the true ICP and the pressure measured via LP. Abnormalities such as Chiari malformations, stenosis, or meningeal disease may lead to differences between true ICP and the pressure collected during LP due to compartmentalization of CSF (39). Such constraints may be circumvented by measuring the optic nerve sheath diameter (ONSD) in relation to the eyeball transverse diameter (ETD), i.e., the ONSD/ETD ratio. The optic nerve is surrounded by CSF, directly reflecting ICP, and multiple studies have shown that the ONSD/ETD ratio correlates well with ICP and can be used in conditions such as intracranial hypertension (IH) (40–42). However, we found no evidence that individuals with high OP had elevated ICP more frequently based on this ratio compared to individuals with low OP. In fact, ONSD/ETD and measures of OP were only weakly correlated in our participants, with a beta coefficient of 0.06. This discrepancy may indicate the presence of CSF compartmentalization, rendering different CSF pressures in different compartments of the brain and spinal canal. Our radiological examinations could not identify any structural abnormalities supporting compartmentalization as discussed earlier. However, these null findings should not be regarded as conclusive. Direct intraventricular measurements could have mitigated this constraint. However, intraventricular measurements were not used due to the method’s invasive nature and ethical concerns. We also did not collect and analyze other data that may influence CSF dynamics, such as osmolality, intracranial venous sinus flow, or CSF production. Such data may aid future studies in identifying explanations for our findings. The strategy for post-LP follow-up could also be improved. While the participants’ subjective data regarding symptomatic improvement post-LP are valuable, using a standardized questionnaire with Likert scales to measure specific symptoms, such as headache, would have provided more reliable data.

There are also several limitations related to the MRI scan assessments. First, the scans were performed with patients in a supine position, which limits the ability to capture certain pathologies related to the spine. Future studies should attempt to use upright MRI, as it will allow clinicians to avoid potential confounders caused by positioning. Second, the scans were collected at a single time point pre-LP, which restricts our ability to determine if symptomatic improvement post-LP was associated with any structural anatomical changes. Third, MRI assessments were made by a senior neuroradiologist and a medical student in cooperation and who were not blinded to each other’s assessment. A future similar study would benefit from having blinded assessors to provide inter-rater reliability ratings of MRI measurements. Despite the above-mentioned limitations, our novel approach raises the question of whether there may be a relationship between CSF pressure, symptomatic relief following LP, and craniocervical anatomy. Further studies on large cohorts of patients and controls are needed to investigate this tentative phenomenon of symptom relief after LP in patients with ME/CFS, which could additionally include radiological examinations using upright MRI.

## Conclusion

This study suggests that a portion of patients with ME/CFS have elevated CSF opening pressures and may experience symptom relief following CSF subtraction. These findings may have clinical implications, with a potentially identifiable subgroup of patients in whom CSF dynamics are of clinical importance. Our data further indicate that certain anatomical structures in the craniocervical area may be related to CSF pressure, although their interpretation remains challenging. These findings indicate the need for further investigation into the complex, multifactorial mechanisms altering the normal physiological flow of CSF and its potential contribution to the disease state of ME/CFS.

## Data Availability

The raw data supporting the conclusions of this article will be made available by the authors, without undue reservation.
